# Supplementary material for: An analysis method of local entropy changes from atomic fluctuations
Source: PLoS One. 2026 Apr 30;21(4):e0347854. doi: 10.1371/journal.pone.0347854 (PMC13132234; doi:10.1371/journal.pone.0347854)
Supplement: Attachment — Submitted filename: Response to Reviewers.docx [file pone.0347854.s002.docx]

Dear Reviewers,

Thank you for your valuable comments. We respond to each point below.

**Reviewer 1**

1. Reviewer comment:
   "First, the authors have made an assumption of independence between atomic phase spaces. ... At very least, the authors should provide a discussion about at what condition this assumption is fulfilled."

Response:
Thank you for this important point. For homopolymers with uniform structure, we consider that the proposed method has no inherent length-scale limitation (e.g., polymer chain length). Nevertheless, as discussed in the manuscript (Figure 5), the phase-space volume of each atom is affected by neighboring particles. When neighboring particles are of the same species, those influences are mutually similar; in that case the method may slightly overestimate some contributions, but we expect the quantitative results to remain reliable as discussed in Figure 5. In contrast, in heterogeneous systems such as copolymers, boundary particles that connect to a different species may have phase-space volumes that differ from particles in continuous same-species regions. Thus, applying the proposed method to strongly heterogeneous materials may require methodological improvements. Since this paper is intended as a first report and a proof-of-concept, we have designated such extensions as future work and added the discussion to the Conclusion section.

2. Reviewer comment:
   "This method is relied on the statistical sampling of atomic fluctuations to compute phase-space volumes. However, the convergence ... is not validated. In particular, the authors should give evidence for convergence at different temperatures.”

Response:

Thank you for your comments. The sampling size and its temperature dependence are discussed in Figure 2. In order to make this clearer we modified the figure title.

2. “Furthermore, the authors have given results in the NVT ensemble. However, the entropy variation due to deformation is sensitive in the NVT ensemble. How the results would be in the NPT or NVE ensemble should be discussed."

Response:
As the reviewer pointed out, our deformation simulations were performed in the NVT ensemble. We confirmed that energy fluctuations during elongation process depend strongly on deformation speed and other simulation conditions. To obtain representative states for entropy evaluation we deform at a fixed rate, then fix the stretch ratio and relax the system until the total energy has converged; sampling is performed from that relaxed, energetically stable state (e.g. Figure 1). Because we sample from these energy-stable states, we expect equivalent results in the NVE ensemble. As an example, the energy history during sampling for the results shown in Figure 4(a). The energy is effectively stable during the sampling window. We have added this explanation and the example energy history to the manuscript.

3. Reviewer comment:
   "The validation of this method in the coarse-grain model is acceptable. I am particularly concerned whether this method could be applied in the all-atom models of other well-studied polymers ... how this model could estimate configuration-related entropy like the torsional entropy."

Response:
Thank you for your comments. As indicated in equations (6) and (7) of the manuscript, by applying appropriate molecular-weight-based weighting the proposed approach can be extended to all-atom models. We have already applied related entropy measurements for polymers at inorganic-material interfaces (work in progress). However, presenting full all-atom results in this first paper would substantially increase the manuscript length and broaden the scope beyond the present proof-of-concept. Therefore, we confined this report to coarse-grained-model results and will present comprehensive all-atom applications, including discussion of torsional/configurational entropy contributions, in a subsequent paper.

**Reviewer 2**

1. Reviewer comment:
   "In Figure 6, the authors compare the distributions of the NoS for dangling chains and network strands at stretch ratios of 1 and 3 ... It would be beneficial ... if the authors could add a brief explanation ... why these specific stretch ratios were selected ... and why others (particularly 2, 4, and 5) are not included."

Response:
Thank you for your comments. As shown in Figure 4, entropic elasticity region is observed up to a stretch ratio of 3. To focus on changes of the NoS within the entropic elasticity region we compared results at stretch ratios 1 and 3. The result at stretch ratio 2 is essentially an interpolation between 1 and 3 and thus was omitted for clarity. We have added this explanatory sentence to the main text.

2. Reviewer comment:
   "Figure 7(a) visualizes the extended polymer chains at a stretch ratio of 5 ... However, the distributions of the NoS shown in Figure 7(b) only cover the range from stretch ratio 1 to 4 ... Please explain why the distribution at stretch ratio 5 is not included in Figure 7(b)."

Response:
Thank you for pointing this out. We have now added the data at stretch ratio of 5 to Figure 7(b). The added result shows the expected further decrease of entropy at that deformation level.

3. Minor corrections (all fixed):
