# Supplementary material for: An analysis method of local entropy changes from atomic fluctuations
Source: PLoS One. 2026 Apr 30;21(4):e0347854. doi: 10.1371/journal.pone.0347854 (PMC13132234; doi:10.1371/journal.pone.0347854)
Supplement: Attachment — Submitted filename: Response_to_Reviewers_auresp_2.docx [file pone.0347854.s003.docx]

Dear Reviewers,

Thank you for your valuable comments. We respond to each point below.

**Reviewer #2: The authors have satisfactorily addressed most of my previous comments.
However, the figure legend for Figure 7 should be amended to align with the revised figure.**

Response: The figure legend for Figure7(b) was changed from “The distributions of the NoS for the extracted atoms constituting the extended chains from a stretch ratio of 1 to a stretch ratio of **4**.” to ““The distributions of the NoS for the extracted atoms constituting the extended chains from a stretch ratio of 1 to a stretch ratio of **5**.”
